# Structural Basis and Selectivity of Tankyrase Inhibition by a Wnt Signaling Inhibitor WIKI4

**DOI:** 10.1371/journal.pone.0065404

**Published:** 2013-06-06

**Authors:** Teemu Haikarainen, Harikanth Venkannagari, Mohit Narwal, Ezeogo Obaji, Hao-Wei Lee, Yves Nkizinkiko, Lari Lehtiö

**Affiliations:** 1 Biocenter Oulu, Department of Biochemistry, University of Oulu, Oulu, Finland; 2 Pharmaceutical Sciences, Department of Biosciences, Abo Akademi University, Turku, Finland; Medical School of Hannover, United States of America

## Abstract

Recently a novel inhibitor of Wnt signaling was discovered. The compound, WIKI4, was found to act through tankyrase inhibition and regulate β-catenin levels in many cancer cell lines and human embryonic stem cells. Here we confirm that WIKI4 is a high potency tankyrase inhibitor and that it selectively inhibits tankyrases over other ARTD enzymes tested. The binding mode of the compound to tankyrase 2 was determined by protein X-ray crystallography to 2.4 Å resolution. The structure revealed a novel binding mode to the adenosine subsite of the donor NAD^+^ binding groove of the catalytic domain. Our results form a structural basis for further development of potent and selective tankyrase inhibitors based on the WIKI4 scaffold.

## Introduction

Tankyrases are enzymes catalyzing a covalent modification of proteins, poly(ADP-ribosyl)ation or PARsylation. In the reaction the enzyme cleaves NAD^+^ to nicotinamide and ADP-ribose, which is then covalently attached to an acceptor protein. Subsequent additions of ADP-ribose units lead to a growing ADP-ribose polymer (PAR) attached to the target protein. Enzymes catalyzing this protein modification and sharing a homologous catalytic domain form a superfamily of 17 members in human (EC 2.4.2.30) [1]. Tankyrase 1 (TNKS1/PARP-5a/ARTD5) and tankyrase 2 (TNKS2/PARP-5b/ARTD6) belong to the polymer forming class of this enzyme family (ARTD1-6), but they have a unique domain organization separating them from the other members. In addition to the catalytic ARTD domain located at the C-terminus, they contain a sterile alpha motif (SAM) next to the catalytic domain, which is responsible for the multimerization of the tankyrases. The target proteins are recognized by five ankyrin repeat clusters (ARC) and the interactions of the ARCs link tankyrases to various cellular pathways [2]. Human tankyrases are highly conserved with 89% sequence identity and share overlapping functions. TNKS1 contains an additional N-terminal region with repeats of histidine, proline, and serine residues, but the function of this motif is so far unknown.

TNKS1 was discovered as an enzyme controlling the length of human telomeres [3] and this was the first implication that tankyrase inhibitors could be useful as therapeutic agents against cancer. Later, TNKS2 was discovered [4] and multiple roles of tankyrases in various cellular signaling pathways have implied that tankyrase inhibitors could be potential drugs especially towards different forms of cancer [5].

The rationale for using tankyrase inhibitors in cancer therapy comes from its various functions within the cell. Tankyrases PARsylate TRF1, a shelterin complex protein protecting telomeres. The modification causes dissociation of TRF1 from the telomeres allowing extension of the telomere by a telomerase enzyme. Due to high telomerase activity, tumor cells escape cellular senescence by uncontrolled telomere extension. Inhibition of tankyrase catalytic activity in tumor cells prevents uncontrolled telomere extension, triggering cellular senescence [3], [6].

Tankyrase 1 is also involved in mitosis as the protein is localized to spindle poles and its catalytic activity is essential for normal bipolar spindle structure [7]. TNKS1 depletion leads to mitotic arrest without DNA damage in HeLa cells [8], while some other cell lines undergo mitosis with subsequent DNA damage and arrest with a senescence-like phenotype [9]. The cellular factors behind these events are poorly understood and remain to be elucidated before the therapeutical potential of tankyrase inhibition in this setting is evaluated.

Wnt signaling pathway is often overactivated in cancers. The identification of tankyrases as part of the β-catenin destruction complex has put tankyrases as one of the promising drug targets regulating Wnt signaling [10]. The central component of the canonical Wnt signaling pathway, the destruction complex, regulates the proteolysis of the downstream effector, β-catenin. When the pathway is not activated, β-catenin is constantly phosphorylated by the destruction complex and subsequently ubiquitinylated and proteolysed. Tankyrases regulate the Wnt pathway by PARsylating Axin, the rate-limiting scaffold protein of the destruction complex, leading to its degradation and activation of Wnt signaling. Inhibition of tankyrases prevents Axin degradation and deactivates Wnt signaling by lowering the levels of β-catenin [10].

**Figure 1:**
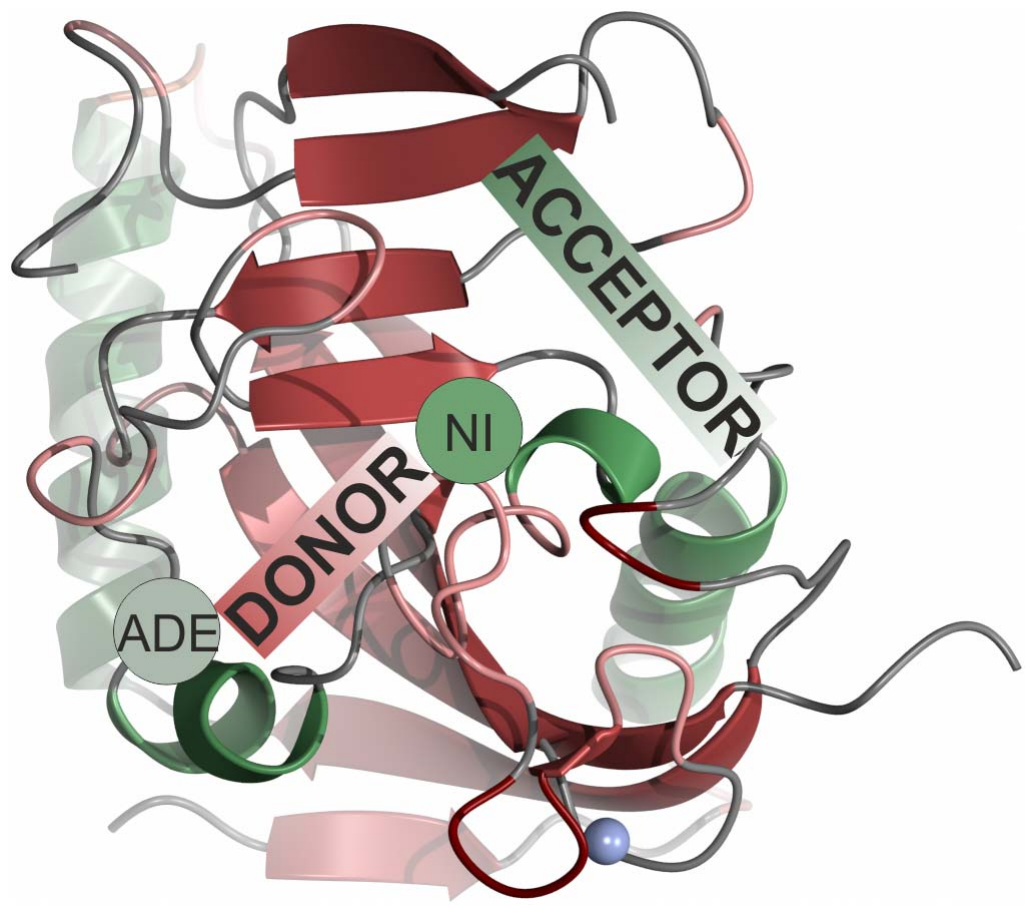
Structure of TNKS2 ARTD domain. Acceptor and donor NAD^+^ binding sites, including nicotinamide subsite (NI) and adenosine subsite (ADE) are labelled.

The first potent tankyrase inhibitor, XAV939, was discovered though the Wnt-responsive luciferase reporter assay [10]. This inhibitor binds to the conserved nicotinamide site of the enzymes [11] and although potent, it is only modestly selective towards tankyrases. Also other inhibitors of tankyrases have been discovered through the inhibition of Wnt-responsive screening [12], [13]. These compounds, IWR-1, JW55, and JW74 do not bind to the conserved nicotinamide subsite of the binding groove, but instead bind to the adenosine subsite of the catalytic domains (Figure 1).

Recently another novel inhibitor of the Wnt signaling pathway, Wnt Inhibitor Kinase Inihibitor 4 or WIKI4, was discovered using β-catenin reporter assays [14]. This small molecule was demonstrated to block Wnt signaling in various cell lines and human embryonic stem cells. It was also demonstrated that WIKI4 inhibited TNKS2 and from a few data points it was estimated that the biochemical IC50 would be as good as 15 nM. WIKI4 is different from the previously characterized TNKS inhibitors and it does not contain a nicotinamide motif present in many ARTD inhibitors [15]. This makes the compound a potential tool as a biological probe for inhibition of tankyrases and Wnt signaling. Its high potency in various cell lines also makes it a potential therapeutic lead compound.

To further characterize the compound we first verified its high potency against TNKS1. We also report the profiling of the compound against many other human ARTD enzymes to verify that the compound is indeed selective for tankyrases over the other ARTD enzymes. Furthermore, we characterized the binding of WIKI4 to the catalytic domain of human TNKS2 using protein X-ray crystallography. This structural work elucidates how the small molecule binds to the protein and explains the selectivity within the ARTD family.

## Methods

### Cloning, Protein Expression and Protein Purification

cDNA for full length ARTD1/PARP1 was purchased from Source Bioscience and the codon optimized full length ARTD2/PARP2 was purchased from Genescript. Full-length ARTD1 was cloned by PCR extension cloning to pNIC28-Bsa4 vector containing an N-terminal 6xHis-tag and a TEV-protease cleavage site (MHHHHHHSSGVDLGTENLYFQ*SM). Full-length ARTD2 was cloned by PCR extension cloning to pNIC28-ZB vector with an N-terminal Z_basic_-tag followed by a 6xHis-tag and a TEV-protease cleavage site (MHHHHHHSSGVDNKFNKERRRARREIRHLPNLNREQRRAFIRSLRDDPSQSANLLAEAKKLNDAQPKGTENLYFQ*SM). Constructs for the catalytic fragments of ARTD4 (residues 250–565) and ARTD12 (residues 489–684) were generous gifts of the SGC Stockholm. These constructs are also based on the pNIC28-Bsa4 vector. Other expression constructs used here have been described before [16]–[18].

### ARTD1/PARP1

ARTD1 plasmid was transformed into *E. coli* Rosetta 2 (DE3) competent cells. An overnight preculture was grown in LB broth medium with 50 µg/mL kanamycin and 34 µg/mL chloramphenicol at 37°C. The preculture was used to inoculate terrific broth auto-induction media (Formedium) with the same antibiotics. The culture was incubated at 37°C with shaking. When OD_600_ reached 0.4–0.5, 0.1 mM ZnSO_4_ was added. Temperature was lowered to 16°C at OD_600_ 1 and the incubation was continued for 16 h. The cells were collected by centrifugation (5020×*g*, 1 hour at 4°C) and the cell pellet was resuspended in 1.5 mL/g lysis buffer (50 mM HEPES, 500 mM NaCl, 10% glycerol, 10 mM imidazole, 0.1% IGEPAL, 0.5 mM TCEP, pH 8.0) and stored at −20°C.

Cell suspension was thawed in warm water and supplemented with 250 U benzonase (Sigma-Aldrich) and 0.1 mM pefabloc (Sigma-Aldrich). Cells were lysed by sonication and the cell debris was cleared by centrifugation (31000×*g*, 45 min at 4°C). Supernatant containing ARTD1 was filtered through 0.45 µm filter and loaded on a HisTrap FF Crude column (GE Healthcare). The column was washed with 40 mL wash buffer 1 (20 mM HEPES, 500 mM NaCl, 10% glycerol, 10 mM imidazole, 0.5 mM TCEP, pH 7.5) followed by 30 mL wash buffer 2 (20 mM HEPES, 1 M NaCl, 10% glycerol, 10 mM imidazole, 0.5 mM TCEP, pH 7.5) and 30 mL wash buffer 3 (20 mM HEPES, 500 mM NaCl, 10% glycerol, 25 mM imidazole, 0.5 mM TCEP, pH 7.5). ARTD1 was eluted with 10 mL of elution buffer (20 mM HEPES, 500 mM NaCl, 10% glycerol, 500 mM imidazole, 0.5 mM TCEP, pH 7.5).

The protein solution was diluted with a buffer so that final concentration of NaCl became 250 mM and loaded to HiTrap Heparin HP column (GE Healthcare). Protein was purified with NaCl gradient elution using a BioLogic Duoflow chromatography system (Bio-Rad). ARTD1 was eluted at 600 mM NaCl. 30 µg of ARTD1 was obtained per 1 L of cell culture.

### ARTD2/PARP2

ARTD2 plasmid was transformed into *E. coli* Rosetta 2 (DE3) competent cells. An overnight preculture supplemented with 50 µg/mL kanamycin and 34 µg/mL chloramphenicol was grown in LB broth medium at 37°C. The preculture was used to inoculate terrific broth auto-induction media (Formedium) supplemented with 0.8% glycerol and the same antibiotics. The culture was incubated at 37°C with shaking until OD_600_ reached 1.5. The temperature was lowered to 18°C and the incubation was continued for 22 hours. The cells were collected by centrifugation (16000×*g*, 20 minutes at 4°C) and the cell pellet was resuspended in 1.5 mL/g lysis buffer (50 mM HEPES, 500 mM NaCl, 10% glycerol, 10 mM imidazole, 0.5 mM TCEP, pH 7.4) and stored at −20°C.

Cell suspension was thawed at room temperature and supplemented with 250 U benzonase (Sigma-Aldrich). Cells were lysed by sonication and the cell debris was cleared by centrifugation (31000×*g*, 45 min at 4°C). Supernatant containing ARTD2 was filtered through 0.45 µm filter and loaded on a 1 ml HisTrap FF Crude column (GE Healthcare). The column was washed with 30 mL of wash buffer (20 mM HEPES, 500 mM NaCl, 10% glycerol, 10 mM imidazole, 0.5 mM TCEP, pH 7.4), followed by 30 mL wash buffer 2 (20 mM HEPES, 500 mM NaCl, 10% glycerol, 25 mM imidazole, 0.5 mM TCEP, pH 7.4). ARTD2 was eluted with 15 mL of elution buffer (20 mM HEPES, 500 mM NaCl, 10% glycerol, 300 mM imidazole, 0.5 mM TCEP, pH 7.4). The 6xHis-tag with the Z_basic_ fragment was cleaved with TEV-protease (1∶30 ARTD2/TEV molar ratio) at 4°C for 24 hours. The protein was then loaded to the 1 mL HisTrap FF Crude column (GE Healthcare) and the flowthrough containing ARTD2 was collected. The yield of ARTD2 was 1 mg per one liter of cell culture.

### ARTD4/PARP-4

ARTD4 construct was transformed into *E. coli* Rosetta 2 (DE3) competent cells. An overnight preculture supplemented with antibiotics (50 µg/mL kanamycin and 34 µg/mL chloramphenicol) was grown in LB medium at 37°C. The preculture was inoculated in terrific broth autoinduction media (Formedium) supplemented with the same antibiotics and glycerol (8 g/L). The culture was grown at 37°C until OD_600_ reached 1 and the incubation was continued overnight at 18°C. The cells were collected by centrifugation (5500×*g,* 10 minutes at 4°C) and the cell pellet was resuspended in lysis buffer (100 mM HEPES, 500 mM NaCl, 10 mM imidazole, 10% glycerol, pH 7.5) and stored at −20°C.

Cell suspension was quickly thawed in warm water and supplemented with 250 U benzonase, Complete, EDTA-free protease inhibitor tablet (Roche) and 0.1 g of lysozyme. Cell suspension was sonicated and the cell debris was cleared by centrifugation (35000×*g*, 20 minutes at 4°C). After filtering the supernatant with 0.45 µm filter, sample was loaded on a HisTrap FF Crude column (GE Healthcare) pre-equilibrated with the loading buffer (20 mM HEPES, 500 mM NaCl, 10% glycerol, 10 mM imidazole, 0.5 mM TCEP, pH 7.5). After loading the sample, the column was washed with the same buffer. Protein was eluted with the elution buffer (loading buffer containing 500 mM imidazole) on Äkta purifier (GE Healthcare). Fractions were pooled together and further purified with Superdex 200 HR 10/30 pre-equilibrated with gel filtration buffer (20 mM HEPES, 500 mM NaCl, 10% glycerol, 0.5 mM TCEP, pH 7.5). 2 mg of ARTD4 was obtained per 1 L of cell culture.

### ARTD12/PARP-12

ARTD12 plasmid was transformed into *E. coli* Rosetta 2 (DE3) competent cells. An overnight preculture was grown in LB medium with 50 µg/mL kanamycin and 34 µg/mL chloramphenicol at 37°C. The preculture was inoculated into terrific broth auto-induction media (Formedium) with the same antibiotics. The culture was incubated at 37°C with shaking. When OD_600_ reached 1 the temperature was lowered to 18°C and the incubation was continued overnight. The cells were collected with centrifugation (5020×*g*, 1 hour at 4°C) and the cell pellet was resuspended in 1.5 mL/g lysis buffer (50 mM HEPES, 500 mM NaCl, 10% glycerol, 10 mM imidazole, 0.5 mM TCEP, pH 7.8) and stored at −20°C.

Cell suspension was thawed in warm water and supplemented with 250 U of benzonase (Sigma-Aldrich) and 1 Complete, EDTA-free protease inhibitor tablet (Roche). Cells were lysed by sonication and the cell debris was cleared by centrifugation (31000×*g*, 1 hour at 4°C). Supernatant containing ARTD12 was filtered through 0.45 µm filter and loaded on a HisTrap FF Crude column (GE Healthcare). The column was washed with 40 mL of lysis buffer followed by 30 mL wash buffer (50 mM HEPES, 500 mM NaCl, 10% glycerol, 25 mM imidazole, 0.5 mM TCEP, pH 7.8). ARTD12 was eluted with 12 mL of elution buffer (30 mM HEPES, 500 mM NaCl, 10% glycerol, 500 mM imidazole, 0.5 mM TCEP, pH 7.5) and concentrated to 0.8 mL. The concentered protein was further purified with HiLoad 16/60 Superdex 75 pg pre-equilibrated with 30 mM HEPES, 300 mM NaCl, 10% glycerol, 0.5 mM TCEP, pH 7.5. 0.1 mg of ARTD12 was obtained per 1 L of cell culture.

### ARTD5/ARTD6/ARTD7/ARTD10/SRPK2

ARTD5/PARP-5, ARTD6/PARP-6, ARTD7/PARP-15, ARTD10/PARP-10, and SRPK2 were expressed and purified as described earlier [16]–[18].

### Homogenous Activity Assay

Assays were conducted as reported earlier [16], [18], [19]. The potency of WIKI4 against TNKS1 was measured using half log dilutions of the inhibitor. The reaction time was set to achieve less than 30% conversion and the assay was done with protein and inhibitor controls to exclude the effect of autofluorescence. Reactions were done in quadruplicates. IC_50_ curves were fitted using sigmoidal dose response curve with four variables using GraphPad Prism version 5.04 for Windows (GraphPad Software). Assay buffer consisted of 50 mM Bis-Tris propane pH 7, 0.5 mM TCEP, and 0.01% Triton-X-100. After the enzymatic reaction 20 µL of 20% acetophenone in ethanol and 20 µL of 2 M KOH were added and the plate was incubated for 10 minutes. After adding 90 µL of formic acid the plate further incubated for 20 minutes and the fluorescence intensity was measured using Tecan Infinity M1000 with excitation/emission wavelengths of 372 nm and 444 nm, respectively.

The profiling assays were carried out similarly, but in order to get a robust answer, the reaction time was set to achieve at least 45% substrate consumption for each enzyme. The reactions were done in triplicates and protein, DMSO, and inhibitor controls were added to exclude the effects of autofluorescence and DMSO on protein activity. The detailed conditions including buffers and incubation times used for the profiling assays are shown in Table 1.

**Table 1:** Conditions used for the profiling of the inhibitors against human ARTDs.

Protein	NAD^+^	Incubation time	Conversion	Buffer
ARTD1 (10 nM)	500 (nM)	40 minutes	68%	50 mM Tris pH 8, 20 µg/mL activated DNA
ARTD2 (400 nM)	500 (nM)	4 hours	61%	50 mM Tris pH 8.0, 0.5 mM TCEP
ARTD4 (400 nM)	500 (nM)	2.5 hours	59%	50 mM Na-phosphate pH 7.5, 0.5 mM TCEP, 1 mg/mL BSA
TNKS1 (200 nM)	500 (nM)	7 hours	46%	50 mM Bis-Tris propane pH 7, 0.5 mM TCEP, 0.01% Triton X-100
TNKS2 (400 nM)	500 (nM)	4 hours	69%	50 mM Bis-Tris propane pH 7, 1 mM TCEP, 2 mM NiCl_2_
ARTD7 (350 nM)+SRPK2 (500 nM)	250 (nM)	2.5 hours	50%	50 mM Na-phosphate pH 7.0
ARTD10 (500 nM)+SRPK2 (2000 nM)	500 (nM)	2.5 hours	79%	50 mM Tris pH 7.0
ARTD12 (1000 nM)	250 (nM)	3 hours	59%	50 mM Na-phosphate pH 7.0

### Crystallization, Data Collection and Refinement

Crystallization of TNKS2 catalytic domain is done as previously reported [17]. 2 mM TCEP and 1∶100 w/w chymotrypsin was added to the protein solution before it was concentrated to 5.8 mg/mL using VIVASPIN 20 concentrators (10 kDa MWCO) (Sartorius Stedim Biotech). Nanodrop (Thermo Fisher Scientific Inc.) was used to measure protein concentration using calculated extinction coefficient of 0.972. The protein was crystallized using a sitting-drop vapor-diffusion method. Equal volumes (100 nl) of well solution (0.2 M LiSO4, 0.1 M Tris-HCl pH 8.5, 24–26% PEG 3350) and protein solution were mixed and incubated at 4°C. Crystals appeared within a week and grew to a size of 50×50×50 µm in one week time. 400 µM WIKI4 was soaked into the crystals in the well solution supplemented with 250 mM NaCl. The crystals were incubated for 24 hours and quickly soaked in a well solution containing 400 µM WIKI4, 20% glycerol, and 250 mM NaCl before flash freezing them in liquid nitrogen.

Diffraction data were collected on beam line I04-1 at Diamond Light source, Didcot, UK. Data were processed and scaled with XDS package [20]. Phases for the structure were directly obtained from the tankyrase 2– nicotinamide complex (PDB accession code 3U9H) [17] and the structure was refined with REFMAC5 [21] and phenix.refine [22]. Coot was used for visualization and manual building of the model [23]. Data collection and refinement statistics are shown in Table 2.

**Table 2:** Data collection and refinement statistics.

PDB code	4BFP
**Data processing**	
Beamline	Diamond I04-1
Wavelength (Å)	0.92
Space goup	C222_1_
Cell dimensions	
a, b, c (Å)	88.96, 93.17, 117.05
Resolution (Å)*	30.0–2.40 (2.46–2.40)
R_merge_ *	0.126 (0.895)
I/σI*	12.7 (2.3)
Completeness (%)*	99.8 (100.0)
Redundancy*	6.8 (7.1)
CC1/2 (%)*	99.7 (77.6)
**Refinement**	
Reflections	19332
R_work_/R_free_	0.202/0.250
B-factors	
Protein	39.7
WIKI4	38.1
Zn	33.5
SO_4_	44.8
Water	37.7
R.m.s.d.	
Bond lengths (Å)	0.002
Bond angles (°)	0.591
Ramachandran plot	
Residues in most favorable regions (%)	97.1
Residues in additionally allowed regions (%)	2.9

* Values for the highest resolution shell are shown in parentheses.

## Results

### Inhibitor Potency

**Figure 2:**
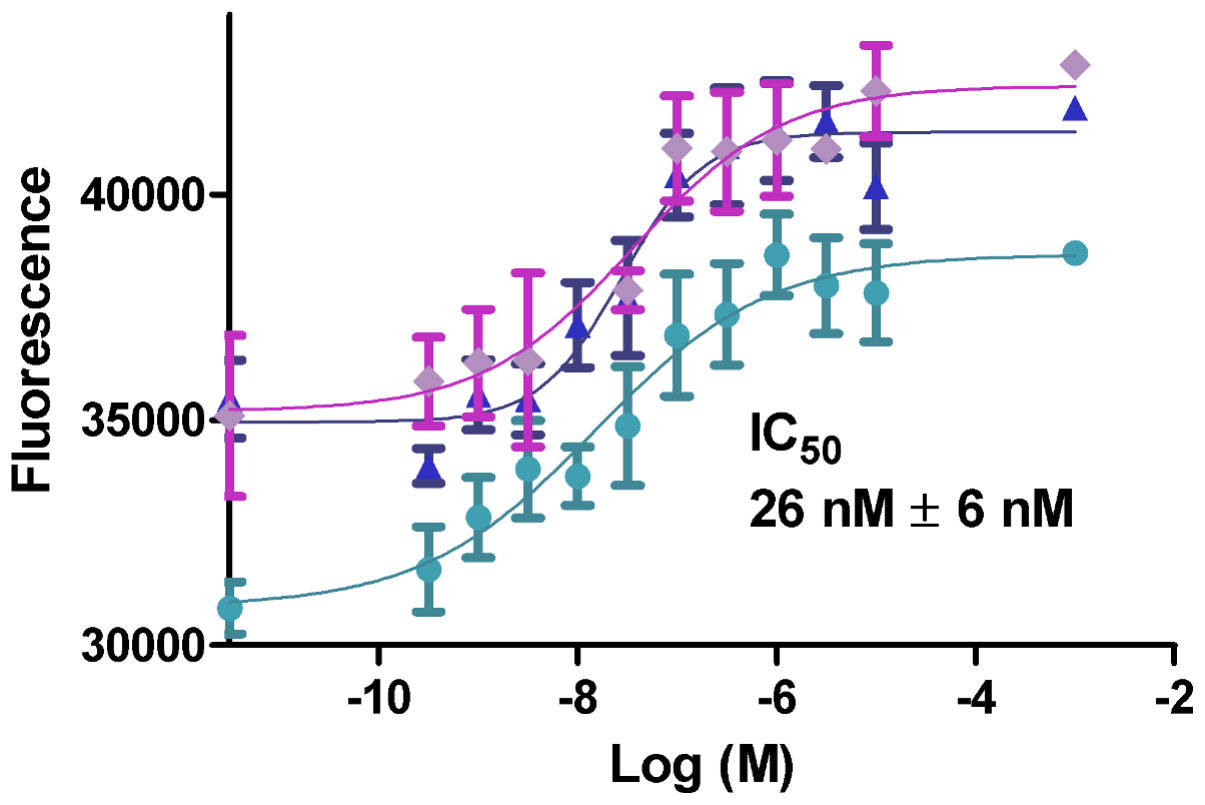
Potency of WIKI4 against TNKS1. The *in vitro* dose response curves were measured three times with a fluorescence-based homogenous activity assay.

The *in vitro* inhibitor potency of WIKI4 to TNKS1 was measured with a fluorescence based activity assay (Figure 2). The IC50 value previously measured for TNKS2 [14] with a few data points showed that the IC50 value of WIKI4 is in the low nanomolar range. IC50 of WIKI4 to TNKS1 is 26 nM, showing that the compound inhibits both isoforms with high potency. When compared to other tankyrase inhibitors, WIKI4 is more potent than IWR-1 (TNKS1 IC_50_ 131 nM) [10] and almost equally potent with XAV939 (TNKS1 IC_50_ 11 nM) [10], a JW74 analog G007-LK (TNKS2 IC_50_ 25 nM) [24], and a recently identified long quinazolinone derivative (TNKS1 IC_50_ 8 nM) [25] binding to both nicotinamide and adenosine subsites.

### Inhibitor Selectivity

**Figure 3:**
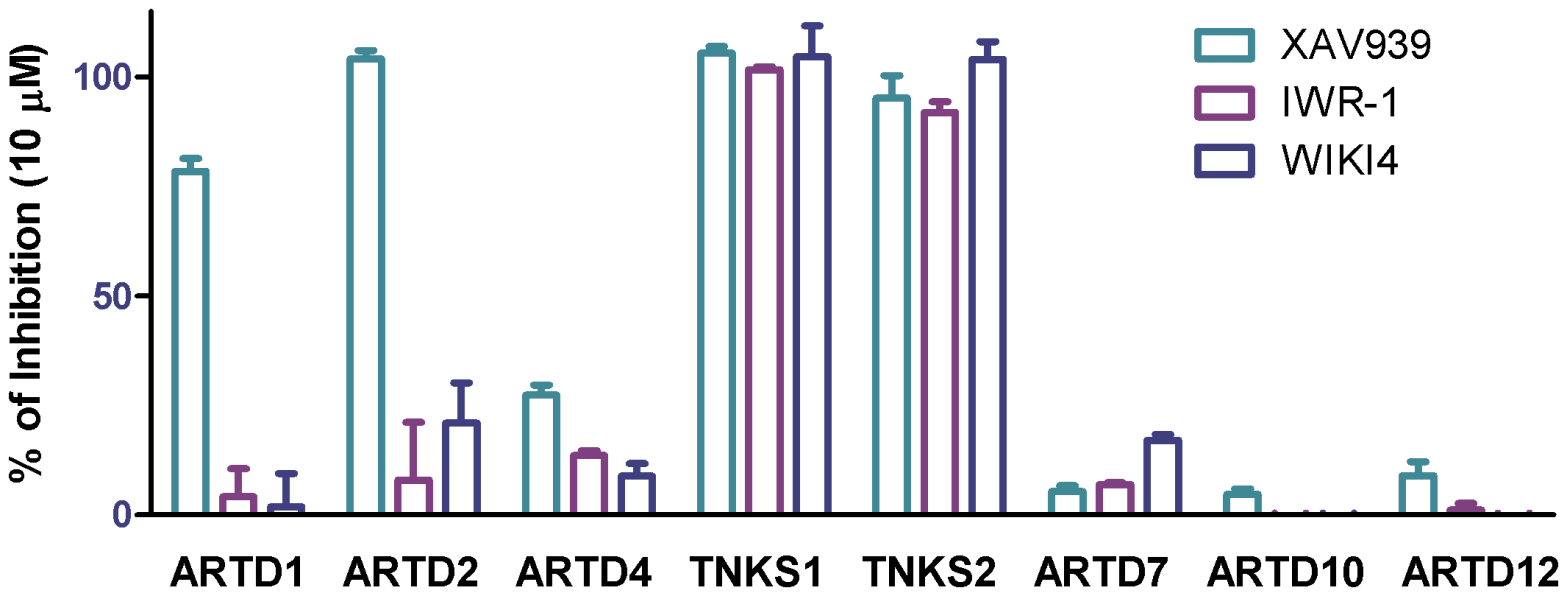
Profiling of inhibitor selectivity. The selectivity of WIKI4 against 8 ARTDs polymerases was measured at 10 µM concentration. XAV939 and IWR-1 were used as controls.

We profiled the inhibition of WIKI4 against 8 ARTDs, including polymerases (ARTDs 1, 2, 4, 5, and 6) and monotransferases (ARTDs 10, 12, and 15) (Figure 3). WIKI4 did not show significant inhibition of other ARTD family members besides tankyrases at 10 µM. As controls we used two known tankyrase inhibitors, XAV939 and IWR-1. IWR-1 also binds to the adenosine site of tankyrases and is selective towards tankyrases, whereas XAV939, which binds to the nicotinamide site inhibits also ARTD1 and ARTD2 (Figure 3). XAV939 has also previously been reported to inhibit ARTD1 and ARTD2, with good potency against ARTD2 (IC_50_ 114 nM) [10].

### WIKI4 Binding

**Figure 4:**
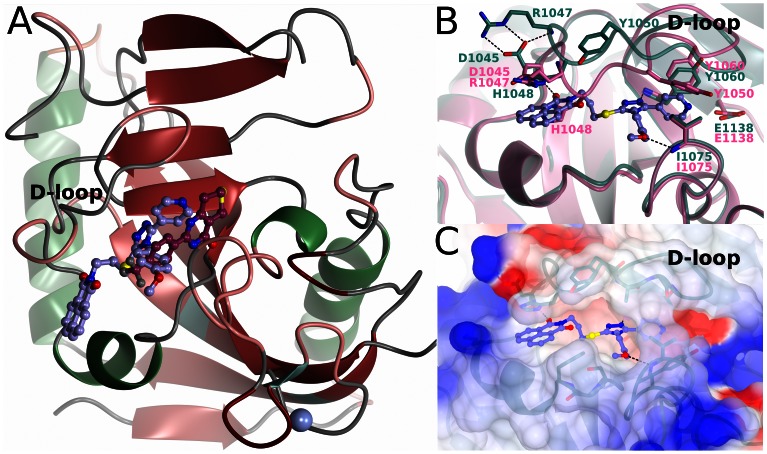
Binding of WIKI4 to TNKS2. a) An overview of TNKS2 structure showing the binding site of WIKI4 (lilac) and XAV939 (dark purple) (pdb accession code 3KR8). b) Comparison of apo TNKS2 structure (pink) (pdb accession code 3KR7) and WIKI4 (turquoise) bound structure of TNKS2. c) Surface electrostatic presentation of WIKI4 binding site. Positive (surface potential charge above 0.25 V) and negative (surface potential charge below −0.25 V) electrostatic regions are colored blue and red, respectively.

The crystal structure of TNKS2-WIKI4 complex was refined to 2.4 Å resolution. The final model is of good quality with R/R_free_ of 20.2%/25.0% (Table 2). Due to lack of electron density the N-terminal residues 946–951, C-terminal Gly1162 and Lys1114, as well as Met1115 located in the loop between strands 6 and 7 were not modeled. There is a nick in the loop between strands 6 and 7 caused by *in situ* proteolysis (see Methods). Importantly the nick does not affect the active site or overall protein structure but aids in crystal packing [11]. Also Ile1051 in the B chain was truncated at Cβ due to lack of electron density. The overall fold of the structure is very similar to TNKS2 apo structure (PDB accession code 3KR7) [11] with a rmsd of 0.59 Å for all Cα atoms. Although the apo and WIKI4 bound TNKS2 structures are overall very similar, major structural rearrangements can be seen in the D-loop lining the active site, as the loop opens to accommodate the inhibitor. In contrast to XAV939 and most other ARTD inhibitors which bind to the nicotinamide subsite of the donor NAD^+^ binding groove, WIKI4 binds mainly to the adenosine subsite (Figure 4a). Both carboxylate oxygens of Asp1045 in the loop form hydrogen bonds with Arg1047 stabilizing the open conformation of the D-loop (Figure 4b). His1048 moves away from the adenosine site (Cα_apo_ – Cα_WIKI4_ 3.8 Å) to accommodate the 1,8-naphthalimide moiety of the inhibitor and Tyr1050 moves out from the binding site (Cα_apo_-Cα_WIKI4_ 5.2 Å) to accomodate the triazole moiety of the inhibitor (Figure 4b). The binding site for WIKI4 is mostly hydrophobic (Figure 4c) and devoid of any water molecules. The compound forms extensive hydrophobic interactions with the binding site, while three hydrogens bonds anchor WIKI4 to the site (details below).

**Figure 5:**
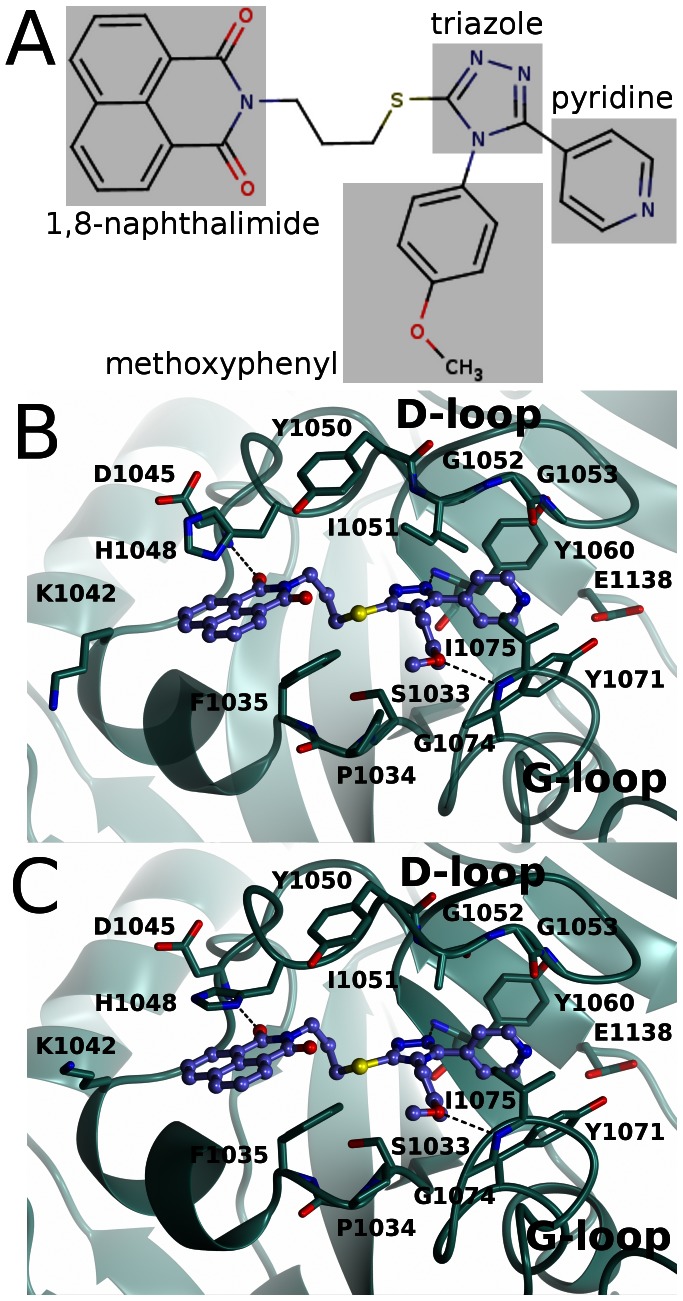
Interactions of WIKI4 with TNKS2 catalytic domain. a) Chemical structure of WIKI4. b) Binding mode of WIKI4 to monomer A. c) Binding mode of WIKI4 to monomer B.

Although WIKI4 does not interact with the nicotinamide site, the pyridine ring of the compound (Figure 5a) is located near the site in a hydrophobic pocket lined by Tyr1060, Tyr1071, Ile1075, Ile1051, and Gly1053 (Figure 5b,c). Nevertheless, it does not form an efficient π-π stacking interaction with Tyr1071 like most other ARTD inhibitors, but it is stacking with Tyr1060 (Figure 5b,c). The amide of the pyridine points outside of the pocket towards the nicotinamide site and is situated in a more polar environment near (3.9 Å) the hydroxyl of Tyr1071 (Figure 5b,c).

The methoxyphenyl group of the compound (Figure 5a) is situated in a mainly hydrophobic pocket formed by Ile1051, Ile1075, Pro1034, Phe1035, and Ser1033 (Figure 5b,c). The oxygen of the methoxy group turns out of the hydrophobic patch of the pocket and forms a hydrogen bond with the backbone amide of Ile1075. An amide of the core triazole moiety (Figure 5a) forms a hydrogen bond with the backbone amide of Tyr1060 (Figure 5b,c).

The 1,8-naphthalimide moiety (Figure 5a) linked to the triazole *via* a flexible spacer seems to mimic the binding of the adenosine of NAD^+^ to the protein. Accordingly, the adenosine moiety of NAD^+^ in Diphteria toxin binds to the same site (pdb accession code 1TOX) [26]. 1,8-naphthalimide moiety is bound between Phe1035 and His1048 and forms a parallel stacking interaction with His1048 (Figure 5c). The stacking interaction has also been observed with other inhibitors binding to this site [17], [24], [27]. However, in the monomer A of the crystal structure, an T-shaped stacking of His1048 with the 1,8-naphthalimide moiety is observed (Figure 5b). This is due to a crystal contact in that monomer, where His1048 stacks with a symmetry-related His1048 and forms a parallel stacking interaction with the residue. In monomer B the conformation of the D-loop is not affected by the crystal symmetry and therefore the conformation observed in this monomer reflects better the situation in solution (Figure 5c). The oxygen of the 1,8-naphthalimide further stabilizes the conformation of the inhibitor by forming a hydrogen bond with the backbone amide Asp1045.

## Discussion

**Figure 6:**
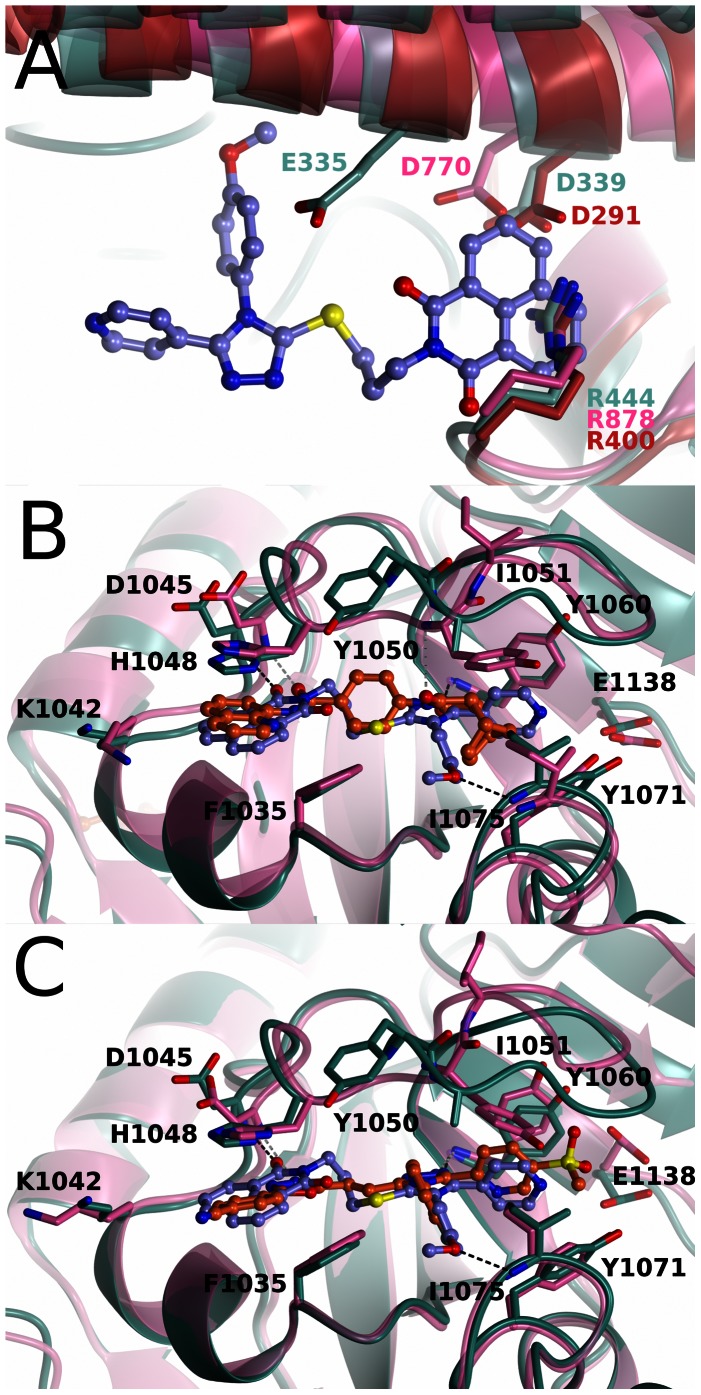
Comparison of WIKI4 binding to tankyrase 2 with other tankyrase selective inhibitors and with ARTD1-3 structures. WIKI4 - TNKS2 protein structure is colored in turquoise and WIKI4 is colored in lilac. a) Comparison of the WIKI4 binding sites in TNKS2, ARTD1 (pink) (pdb accession code 3GJW), ARTD2 (green) (pdb accession code 3KCZ), and ARTD3 (red) (pdb accession code 3FHB). ARD, ARTD regulatory domain. b) Comparison of the binding of WIKI4 and IWR-1. Hydrogen bonds for WIKI4 and IWR-1 are shown in black and gray dotted lines, respectively. IWR-1 - TNKS2 protein structure is colored in pink and IWR-1 is colored in orange. c) Comparison of the binding of WIKI4 and G007-LK. Hydrogen bonds for WIKI4 and G007-LK are shown in black and gray dotted lines, respectively. G007-LK - TNKS2 protein structure is colored in pink and G007-LK is colored in orange.

WIKI4 is a new, recently reported inhibitor scaffold for tankyrases with high potency towards both TNKS isoforms. IC50 towards TNKS1 is 26 nM and the inhibitor profiling showed that WIKI4 is equally potent towards TNKS2 (Figure 3). It also displays high selectivity over other ARTDs as it does not significantly inhibit any of the 6 other ARTDs tested at 10 µM concentration. The selectivity of the compound comes from a few key differences between the catalytic domains of ARTDs. Mainly from interactions with His1048 and Phe1035 which are unique for tankyrases [24]. His1048 forms a stacking interaction with 1,8-naphthalimide moiety and Phe1035 interacts with both the 1,8-naphthalimide and methoxyphenyl parts of the compound (Figure 5b,c). Several ARTDs contain a regulatory domain on the N-terminal side of the ARTD domain and this domain interacts with the donor NAD^+^ binding site. The large 1,8-naphthalimide moiety extends out of the adenosine binding site and clashes with the regulatory domains of ARTD1-3 (Figure 6a) [28]–[30]. Also the methoxyphenyl group extends in the direction of the G-loop, towards the regulatory domains of ARTD1-3, clashing especially in ARTD2 (Figure 6a).

The structure-activity studies of WIKI4 analogs by James and co-workers [14] can be explained by our structural data. The reduced potency resulting from the deletion of the methoxy group (Figure 5a) from the methoxyphenyl moiety disrupts the hydrogen bond made by the methoxy oxygen (Figure 5b,c). The reduction in potency by moving the amide from para to meta position in the pyridyl ring leads to an unfavourable interaction with the hydrophobic residues lining the binding pocket (Figure 5b,c). The methoxy substitutions in the pyrimidyl ring at 1′ and 2′ positions decrease the potency by increasing the size of the moiety leading to steric clashes with surrounding residues, especially with Tyr0171. Similar effect can be seen when pyridyl group is replaced by a 1,3-benzodioxole. Modifications that include the deletion of the methoxyphenyl group inactivate the compound demonstrating also the importance of the hydrophobic interactions of the group for the activity of the compound. Modifications that reduce the size of the 1,8-naphthalimide moiety result in the inactivation of the compound, as does the substitution of 1,8-naphthalimide with a phthalimide group, possibly through the disruption of the stacking interaction with His1048 and a subsequent conformational change of the moiety disrupting the hydrogen bond to Asp1045.

Both WIKI4 and IWR-1 bind to the adenosine site of TNKS2. Despite that both compounds were soaked to preformed crystals and induce the opening of the D-loop, they show completely different conformation of the loop (Figure 6b). This indicates a large structural plasticity of the loop. WIKI4 contains a flexible linker between the triazole and 1,8-naphthalimide groups instead of the benzene ring found in IWR-1. IWR-1 forms two similar hydrogen bonds to the backbone amides of Asp1045 and Tyr1060 as WIKI4. Also His1048 in both structures stack with the compound. The norbornyl ring of IWR-1 does not extend as deeply towards the nicotinamide pocket as the pyridine ring in WIKI4. The binding of WIKI4 also does not result in the rotation of Tyr1071 interacting with the norbornyl moiety of IWR-1. IWR-1 does not extend towards the G-loop, and lacks the interactions made by the methoxyphenyl group of WIKI4 with the loop.

WIKI4 and JW74 analog G007-LK both contain a core triazole moeity linking three groups together. However, the binding of G007-LK induces similar structural changes in the D-loop as the binding of IWR-1. The conformation of His1048, which allows the parallel π-π stacking with WIKI4 and IWR-1 is present also in G007-LK, where it forms a parallel π-π stacking with the benzylamine group (Figure 6c). The conformations of Tyr1050 and Ile1051 are similar in IWR-1 and G007-LK, whereas the D-loop in WIKI4 assumes completely different conformation in this region. WIKI4 contains a longer linker between the triazole cores and the 1,8-naphthalimide/oxadiazole-benzonitrile moieties of the compounds than G007-LK. The longer linker in WIKI4 results in a binding where the triazole and 1,8-naphthalimide groups are not exactly parallel.

The identification of WIKI4 as a Wnt signaling antagonist and its high potency in several cell lines made it a promising hit compound as a tankyrase inhibitor [14]. We have here verified the high potency of the compound and showed that it is highly selective towards tankyrases over other ARTDs. Furthermore, the unique binding mode of the compound to the adenosine site and interactions with the residues lining the donor NAD^+^ binding groove explain both the high potency and selectivity of the compound. As such the compound is an excellent biological probe for the evaluation of the effects of tankyrase inhibition in different cancer cell lines. The structural characterization of the binding mode may also help in developing metabolic stability and pharmacokinetics of WIKI4 in the future.

### Conclusions

We have analyzed the binding mode, potency and selectivity of WIKI4, a novel Wnt antagonist that acts through tankyrase inhibition. WIKI4 is selective for tankyrases over 6 other ARTDs tested and inhibits tankyrases with high potency. The inhibitor binds to the adenosine binding site of tankyrases and has a distinct binding mode compared to other inhibitors binding to this site. The compound uniquely extends towards and interacts with the G-loop, and retains critical hydrogen bonding and stacking interactions as observed in other TNKS inhibitors. Our results form a structural basis for the high potency and selectivity of WIKI4 and allow further rational development of WIKI4 based inhibitors.
